# Involvement of Senescence and Mitochondrial Fission in Endothelial Cell Pro-Inflammatory Phenotype Induced by Angiotensin II

**DOI:** 10.3390/ijms21093112

**Published:** 2020-04-28

**Authors:** Masashi Miyao, Stephanie Cicalese, Tatsuo Kawai, Hannah A. Cooper, Michael J. Boyer, Katherine J. Elliott, Steven J. Forrester, Ryohei Kuroda, Victor Rizzo, Tomoki Hashimoto, Rosario Scalia, Satoru Eguchi

**Affiliations:** 1 Cardiovascular Research Center, Lewis Katz School of Medicine at Temple University, 3500 N. Broad Street, Philadelphia, PA 19140, USA; miyaom@fp.med.kyoto-u.ac.jp (M.M.); tug51315@temple.edu (S.C.); tuf88636@temple.edu (T.K.); tug05907@temple.edu (H.A.C.); tue89415@temple.edu (M.J.B.); kelliott@temple.edu (K.J.E.); steven.forrester@emory.edu (S.J.F.); tun26594@temple.edu (R.K.); rizzov@temple.edu (V.R.); 2 Department of Forensic Medicine, Kyoto University Graduate School of Medicine, Yoshida-Konoe-cho, Sakyoku, Kyoto 606–8501, Japan; 3 Department of Neurosurgery and Neurobiology, Barrow Aneurysm and AVM Research Center, Barrow Neurological Institute, Phoenix, AZ 85013, USA

**Keywords:** endothelial cells, angiotensin II, senolytic, ER stress, inflammation

## Abstract

Angiotensin II (AngII) has a crucial role in cardiovascular pathologies, including endothelial inflammation and premature vascular aging. However, the precise molecular mechanism underlying aging-related endothelial inflammation induced by AngII remains elusive. Here, we have tested a hypothesis in cultured rat aortic endothelial cells (ECs) that the removal of AngII-induced senescent cells, preservation of proteostasis, or inhibition of mitochondrial fission attenuates the pro-inflammatory EC phenotype. AngII stimulation in ECs resulted in cellular senescence assessed by senescence-associated β galactosidase activity. The number of β galactosidase-positive ECs induced by AngII was attenuated by treatment with a senolytic drug ABT737 or the chemical chaperone 4-phenylbutyrate. Monocyte adhesion assay revealed that the pro-inflammatory phenotype in ECs induced by AngII was alleviated by these treatments. AngII stimulation also increased mitochondrial fission in ECs, which was mitigated by mitochondrial division inhibitor-1. Pretreatment with mitochondrial division inhibitor-1 attenuated AngII-induced senescence and monocyte adhesion in ECs. These findings suggest that mitochondrial fission and endoplasmic reticulum stress have causative roles in endothelial senescence-associated inflammatory phenotype induced by AngII exposure, thus providing potential therapeutic targets in age-related cardiovascular diseases.

## 1. Introduction

The vascular endothelium is critical to preserve vascular homeostasis, which includes tonus regulation, barrier function, and anti-coagulation. Thus, endothelial cells (ECs), which form the monolayer of vascular endothelium, are believed to protect against vascular pathology associated with hypertension as well as atherosclerosis, the two major causes for cardiovascular mortality [1,2]. However, ample evidence suggests that the disruption of EC homeostasis, termed endothelial dysfunction, is the predominant driving force for the development of these diseases in human as well as established animal models [1,2,3]. The phenotype of endothelial dysfunction is characterized by reduced vasodilation, disruption in barrier integrity, oxidative stress, and enhanced inflammatory responses. Specifically, the endothelial inflammatory responses involve increased cytokine and chemokine production as well as expression of cell adhesion molecules leading to recruitment of the activated leukocytes to the endothelium [1,2]. However, detailed molecular mechanisms causing the pro-inflammatory phenotype of the dysfunctional ECs are not fully explored, which hampers the development of specific medications to preserve endothelial function.

While renin angiotensin system (RAS) is a physiologically important endocrine system to maintain blood pressure and volume, systemic and local over-reactivity of this system significantly contributes to the development of cardiovascular diseases [4,5]. Accordingly, inhibitors of the RAS, including the antagonists for the angiotensin II type 1 receptor (AT1R), appear effective at protecting against endothelial dysfunction in clinical studies [6] as well as endothelial inflammation in preclinical studies [5,7]. Importantly, the endothelial inflammatory responses can be simulated in vitro with cultured ECs and angiotensin II (AngII) stimulation. Analyses utilizing cultured ECs have provided useful information about the signaling mechanism of EC inflammation via the AT1R stimulation [5,8]. One of the attractive mechanisms which appears to mediate the pro-inflammatory phenotype of ECs in response to AngII is mitochondrial dysfunction, such as an increase in the generation of reactive oxygen species (ROS) [9]. Analogous to endothelial dysfunction, mitochondrial dysfunction is associated with almost all types of cardiovascular disease [10,11]. Although the detailed molecular mechanism by which mitochondrial dysfunction occurs in ECs remains unclear, dysfunctional mitochondria in ECs are frequently associated with more fragmented structures caused by fission [12,13,14]. A GTPase Dynamin-related protein 1 (Drp1) is a master-regulator of mitochondrial fission [15]. It has been recently reported that AngII increases mitochondrial ROS production in cultured vascular smooth muscle cells (VSMCs), which was attenuated by a small molecule inhibitor of Drp1, mitochondrial division inhibitor-1 (mdivi1) [16]. However, limited information is available in ECs regarding the relationship among AngII, mitochondrial fission, and inflammation.

Aging is a nonmodifiable risk factor for almost all types of cardiovascular pathologies, including the development of endothelial dysfunction in humans [17]. Importantly, there are individuals in which accelerated vascular aging has been identified phenotypically, among the predisposed middle-aged populations [18]. However, the molecular mechanism of this prematurely occurring endothelial aging remains obscure. In young rodent models of AngII infusion, vascular cells were prematurely aged, as qualified through signatures of senescence, including increased senescence-associated β galactosidase activity [19,20,21]. It should be noted that the premature cardiovascular aging induced by AngII is associated with disruption of proteostasis, causing endoplasmic reticulum (ER) stress and accumulation of protein aggregates [22,23,24,25]. Important to the present study, AngII stimulation in cultured aortic VSMCs or ECs recapitulates these senescent characteristics of the vasculature [19,26].

In addition to its importance in organ development, senescence is considered as a primary protection mechanism to retard dysfunctional cell phenotypes in over-replicated or over-stressed cells to prevent cancer and apoptosis, respectively [27]. However, senescent cells are not entirely silent in cellular functions, and instead, they alter surrounding cells and tissues via production of specific paracrine factors termed senescence-associated secretory phenotype (SASP), which transmit a pro-inflammatory phenotype to surrounding non-senescent cells [28]. Accordingly, senescence and SASP have been implicated in vascular inflammatory diseases, including atherosclerosis, hypertension, and abdominal aortic aneurysms [29]. Elimination of senescent cells by drugs that cause selective apoptosis of senescent cells appears effective to prevent the development of atherosclerosis in a mouse model [30]. However, cell type-specific roles of senescence, such as those in endothelial inflammation, have not been sufficiently explored.

According to the above literature suggesting the importance of the RAS, mitochondrial fission, and senescence in EC inflammation, we have tested a hypothesis in cultured aortic ECs that removal of senescent cells, preservation of proteostasis, or inhibition of mitochondrial fission attenuates the pro-inflammatory EC phenotype induced by AngII. Our findings support the role of mitochondrial fission and ER stress in mediating senescence and subsequent EC inflammation and thus suggest the need for further investigation of this novel cascade in animal models of cardiovascular diseases and in humans.

## 2. Results

### 2.1. Induction of Senescence by AngII in ECs

**Figure 1:**
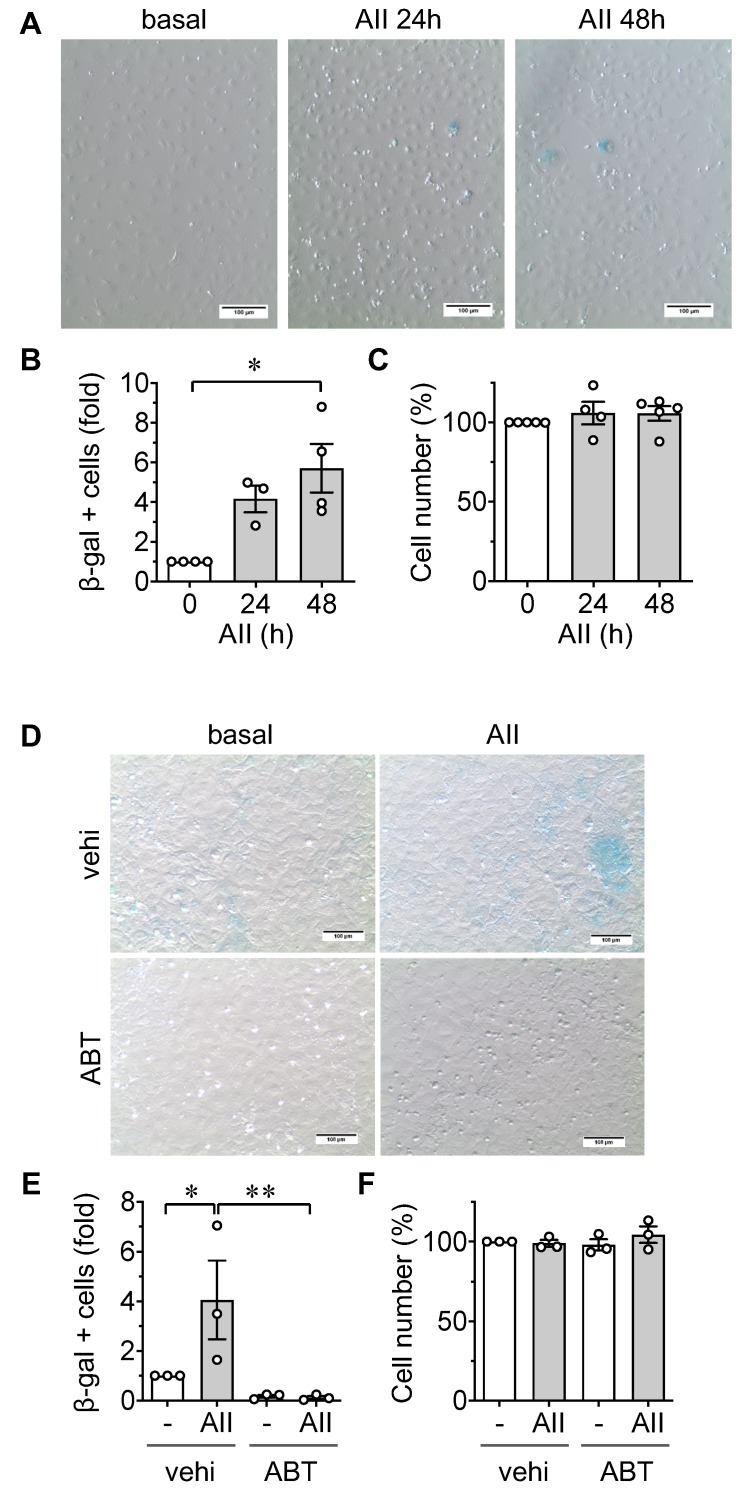
Induction of senescence by angiotensin II (AngII) in endothelial cells. (**A**–**C**) Cultured rat aortic endothelial cells (ECs) were serum-starved for 24 h. ECs were stimulated with 100 nM AngII (AII) for 24 h or 48 h, as indicated. Representative staining data are shown (**A**). Scale bar indicates 100 μm. β galactosidase positive cells (**B**) and total attached cells (**C**) per high power field (HPF) were counted for each group and expressed as fold basal or % basal, respectively. The bars in the graphs show the mean ± SEM from independent experiments (**B**, 0 h *n* = 4, 24 h *n* = 3, 48 h *n* = 4, **C**, 0 h *n* = 5, 24 h *n* = 4, 48 h *n* = 5). 0.54 ± 0.37% were β galactosidase positive in 0 h ECs. (**D**–**F**) ECs pretreated with a senolytic drug, ABT737 (ABT, 30 nM), or vehicle (vehi, 0.1% DMSO final) for 30 min were stimulated with 100 nM AngII (AII) for 48 h. Representative staining data are shown (**D**). Scale bar indicates 100 μm. β galactosidase positive cells (**E**) and total attached cells (**F**) were counted in each group and expressed as fold basal or % basal, respectively. The bars in the graphs show the mean ± SEM from three independent experiments. 1.69 ± 1.20% were β galactosidase positive in basal vehicle ECs. * indicates *p* < 0.05. ** indicates *p* < 0.01.

After stimulation with AngII for 48 h, rat aortic ECs demonstrated an increase in senescence-associated β galactosidase positive cells (Figure 1A,B). Stimulation with AngII did not significantly alter the attached cell numbers (Figure 1C). To verify that the β galactosidase positive cells specifically represent senescent cell populations, rat aortic ECs were co-treated with a senolytic, ABT737, which specifically causes apoptosis of senescent cells [31], or its vehicle dimethyl sulfoxide (DMSO). Treatment with ABT737 almost completely attenuated β galactosidase positive cell populations in ECs induced by the AngII stimulation (Figure 1D,E and Supplementary Figure S1a–c) supporting the specificity of the β galactosidase staining as a marker of senescent ECs. The lack of decline in cell numbers in ABT737 treated ECs (Figure 1F) could be due to the pretreatment of senolytic on non-senescent ECs instead of post-treatment on existing senescent ECs.

**Figure 2:**
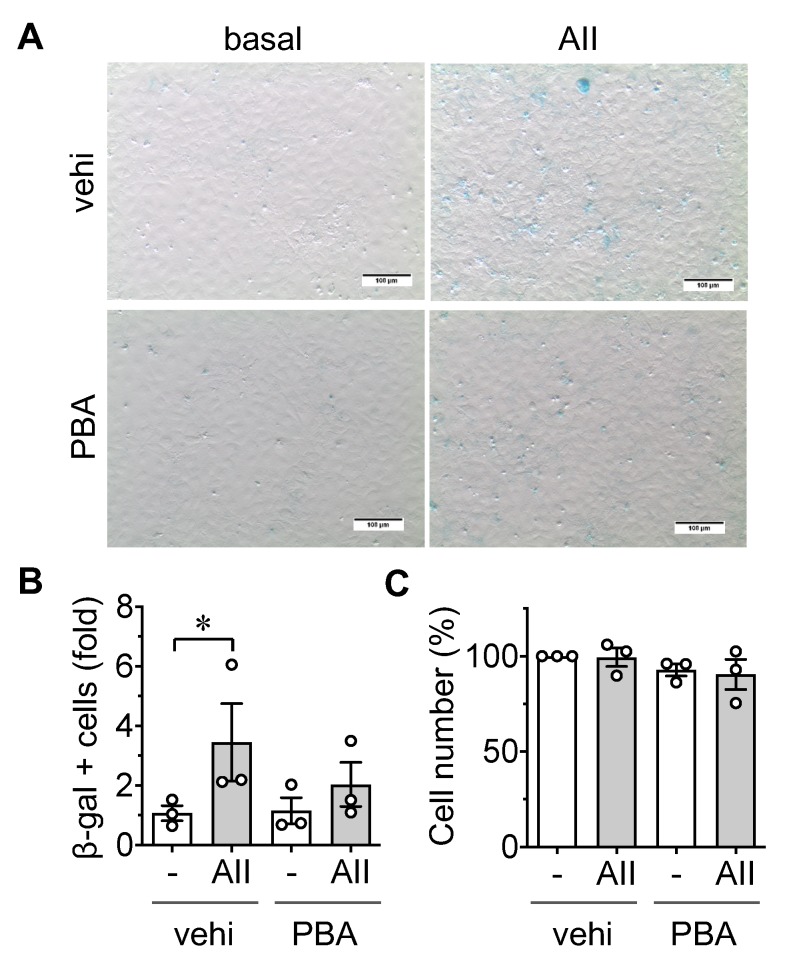
Chemical chaperone mitigates AngII-induced senescence. (**A**,**B**) Rat aortic ECs pretreated with chemical ER chaperone, 4-PBA (PBA, 1 mM), or vehicle (vehi, PBS 0.1% final) were stimulated with 100 nM AngII (AII) for 48 h. Representative staining data are shown (**A**). Scale bar indicates 100 μm. β galactosidase positive cells (**B**) and total attached cells (**C**) were counted in each group and expressed as fold basal or % basal, respectively. The bars in the graphs show the mean ± SEM from three independent experiments. 1.07 ± 0.44% were β galactosidase positive in basal vehicle ECs. ** indicates *p* < 0.01.

AT1R has been implicated in the pathological functions of AngII, including the induction of senescence [5,19]. The disruption of proteostasis, including enhanced protein misfolding, is a potential mechanism by which AT1R mediates senescence [32]. To determine if increased protein misfolding is required for AngII-induced senescence, rat aortic ECs were pretreated with a chemical ER chaperone, 4-phenylbutyrate (4-PBA). Indeed, 4-PBA attenuated AngII induction of senescence in rat aortic ECs without altering the attached cell numbers (Figure 2A–C and Supplementary Figure S1d–f).

### 2.2. Induction of Leukocyte Adhesion via ER stress and Senescence in ECs

**Figure 3:**
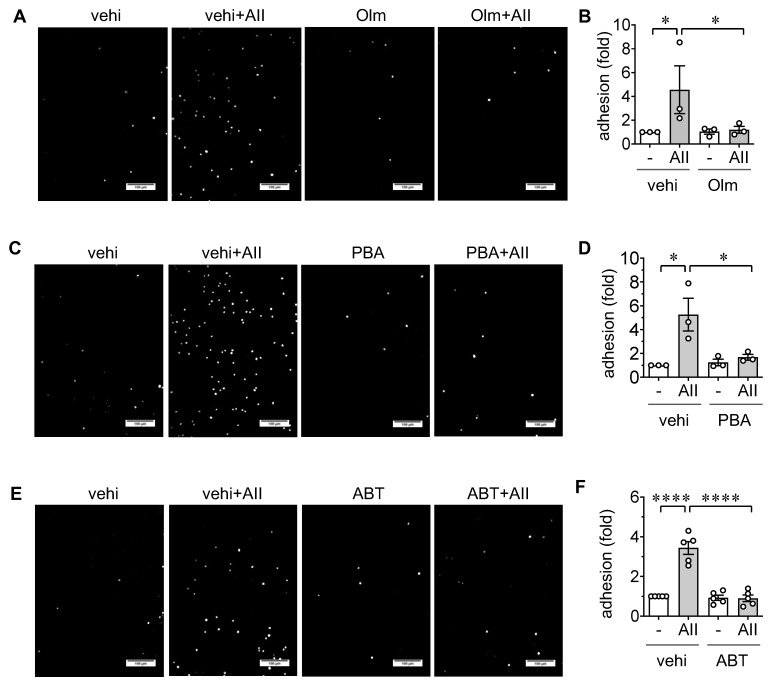
Involvement of angiotensin II type 1 (AT1) receptor, ER stress, and senescence in monocyte adhesion induced by angiotensin II in endothelial cells. (**A**,**B**) Serum starved rat aortic ECs pretreated with AT1R antagonist, Olmesartan (Olm, 10 μM) for 30 min were incubated with 100 nM AngII (AII) for 48 h. ECs were then incubated with THP-1 cells for 30 min, washed, and adherent THP-1 cells were quantified. Representative staining data are shown (**A**). Scale bar indicates 100 μm. Attached THP-1 cells were counted in each group and expressed as fold basal (**B**). 7.7 ± 2.9 THP-1 cells were attached per HPF in basal vehicle ECs. (**C**,**D**) Serum starved ECs pretreated with 1 mM 4-PBA (PBA) for 30 min were incubated with 100 nM AngII (AII) for 48 h. ECs were then incubated with THP-1 cells for 30 min, washed, and adherent THP-1 cells were quantified. Representative staining data are shown (**C**). Scale bar indicates 100 μm. Attached THP-1 cells were counted in each group and expressed as fold basal (**D**). 14.4 ± 10.9 THP-1 cells were attached per HPF in basal vehicle ECs. (**E**,**F**) Serum starved ECs pretreated with ABT737 (ABT, 30 nM) or vehicle (vehi, 0.1% DMSO final) for 30 min were incubated with 100 nM AngII (AII) for 48 h. ECs were then incubated with THP-1 cells for 30 min, washed, and adherent THP-1 cells were quantified. Representative staining data are shown (**E**). Scale bar indicates 100 μm. Attached THP-1 cells were counted in each group and expressed as fold basal (**F**). 5.7 ± 3.3 THP-1 cells were attached per HPF in basal vehicle ECs. The bars in the graphs show the mean ± SEM from three–five independent experiments. * indicates *p* < 0.05. **** indicates *p* < 0.0001.

To assess if AngII induced EC senescence is accompanied by a pro-inflammatory EC phenotype, a THP-1 monocyte adhesion assay was performed. The stimulation of rat aortic ECs with AngII for 48 h significantly increased the adhesion of THP-1 cells. Pretreatment with the AT1R antagonist Olmesartan mitigated the adhesion response (Figure 3A,B and Supplementary Figure S1g–i), verifying that AT1R promotes a pro-inflammatory EC phenotype. As expected, 4-PBA, as well as ABT737, attenuated THP1 adhesion to ECs induced by AngII (Figure 3C–F) suggesting the regulatory roles of ER stress and premature EC senescence in the pathological endothelial inflammation.

### 2.3. Role of Mitochondrial Fission in EC Senescence and Inflammation

**Figure 4:**
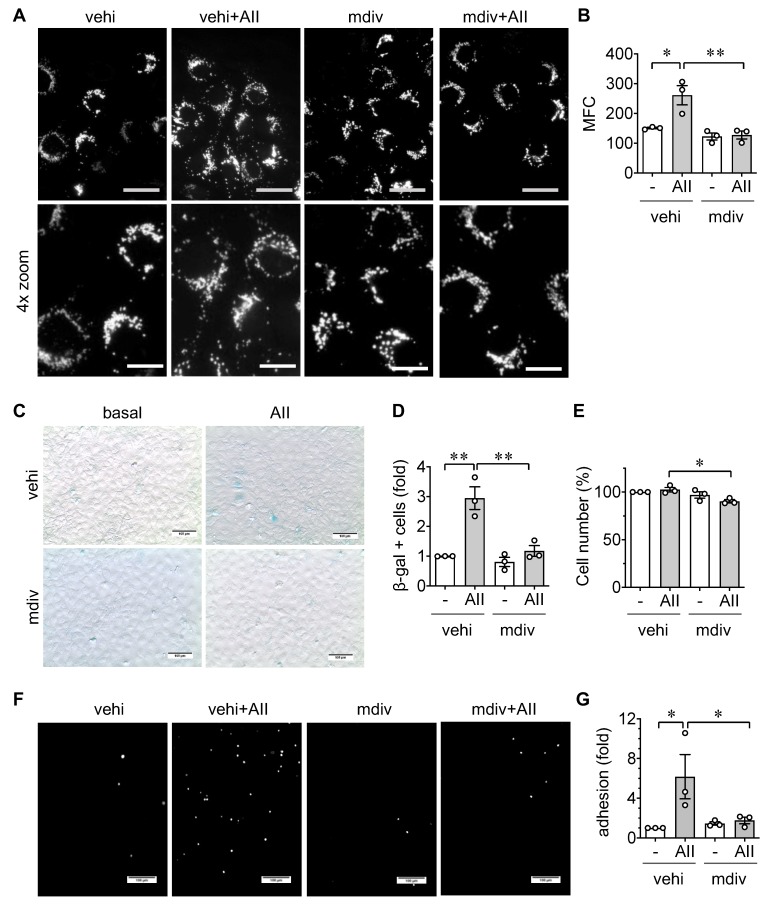
Upstream role of mitochondrial fission in endothelial cell senescence and inflammation induced by AngII. (**A**,**B**) Serum starved rat aortic ECs transduced with 10 moi adenovirus encoding mito-dsRed for 48 h and pretreated with mitochondrial fission inhibitor, mdivi1 (mdiv, 5 μM) or vehicle (vehi, DMSO 0.1% final) for 60 min were stimulated with 100 nM AngII (AII) for 3 h. Representative staining data are shown, and 4x zoomed pictures are included (**A**). Scale bar is 15 μm and 7.5 μm (zoom), respectively. Mitochondrial fission count (MFC) was measured (**B**). (**C**–**E**) Rat aortic ECs pretreated with mdivi1 (mdiv, 5 μM) or vehicle (vehi, DMSO, 0.1% final) for 60 min were stimulated with 100 nM AngII (AII) for 48 h. Representative staining data are shown (**C**). Scale bar indicates 100 μm. β galactosidase positive cells (**D**) and total attached cells (**E**) were counted in each group and expressed as fold basal or % basal, respectively. 0.48 ± 0.17% were β galactosidase positive in basal vehicle ECs. (**F**,**G**) Serum starved rat aortic ECs pretreated with mdivi1 (mdiv, 5 μM) or vehicle (vehi, DMSO 0.1% final) for 60 min were incubated with 100 nM AngII (AII) for 48 h. ECs were then incubated with THP-1 cells for 30 min, washed, and adherent THP-1 cells were quantified. Representative staining data are shown (**F**). Scale bar indicates 100 μm. Attached THP-1 cells were counted in each group and expressed as fold basal (**G**). 14.8 ± 10.1 THP-1 cells were attached per HPF in basal vehicle ECs. The bars in the graphs show the mean ± SEM from three independent experiments. * indicates *p* < 0.05. ** indicates *p* < 0.01.

Mitochondrial fission is frequently seen in EC models of cardiovascular diseases and associated with mitochondrial dysfunction [12,13,14]. Additionally, the contribution of mitochondrial fission in EC senescence has been demonstrated [33]. Accordingly, mitochondrial fission was assessed using mitochondrial fragmentation count (MFC) with or without AngII stimulation for 3 h in rat aortic ECs. AngII stimulated mitochondrial fission in rat aortic ECs. Pretreatment with mdivi1, a pharmacological inhibitor of mitochondrial fission inducer Drp1 [34], attenuated mitochondrial fission induced by AngII (Figure 4A,B). While mdivi1 slightly decreased cell number when combined with AngII, pretreatment with mdivi1 also attenuated AngII-induced senescence and leukocyte adhesion in rat aortic ECs (Figure 4C–G) suggesting that mitochondrial fission contributes to an inflammatory aging phenotype in ECs.

## 3. Discussion

**Figure 5:**
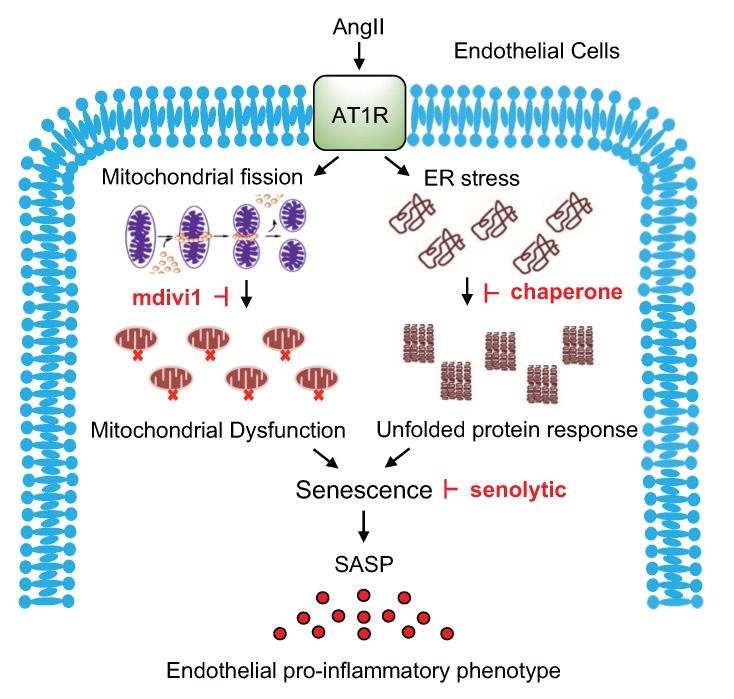
Proposed mechanisms of angiotensin II (AngII)-induced endothelial cell senescence and cardiovascular disease progression. AngII via AngII type 1 receptor (AT1R) induces mitochondrial fission and ER stress in ECs, which leads to EC senescence and inflammation. Inhibitors of mitochondrial fission and ER stress, as well as senolytics could be useful to prevent cardiovascular disease progression via inhibition of senescence-associated endothelial proinflammatory phenotype, including senescence-associated secretory phenotype (SASP).

In the present study, we have demonstrated that AngII induces mitochondrial fission and ER stress in ECs, contributing to EC senescence and pro-inflammatory phenotype (Figure 5). Thus, our data suggest mitochondrial fission as a unique target for therapy to combat aging-related cardiovascular diseases associated with chronic inflammatory conditions.

Accumulating literature suggests the importance of VSMC senescence in AngII-related cardiovascular pathology [5,35]. However, the endothelial concept of AngII-mediated premature cardiovascular aging has been under-developed. Senescent cells actively produce pro-inflammatory cytokines known as SASP. Prior studies have shown the importance of EC SASP in aging related inflammation and cardiovascular pathology [36,37]. We further demonstrated that the elimination of senescent cells prevented leukocyte adhesion to ECs in the present study. As only a few percent of ECs become senescent upon AngII stimulation, it is likely that AngII promotes monocyte adhesion to ECs via SASP.

Endothelial ER stress and unfolded protein response (UPR) have been shown to contribute to atherosclerosis [38]. We and others have shown that AngII infusion caused vascular ER stress and that 4-PBA attenuated UPR and vascular remodeling in rodent models of AngII-induced hypertension [23,24]. The present study adds a role for AngII-induced ER stress in mediating EC senescence and inflammatory phenotype. In human umbilical vein ECs, inhibition of autophagy accelerated AngII-induced senescence [39], further supporting the disruption of proteostasis as a contributor to EC senescence. However, an increasing body of evidence suggests a potentially interdependent relationship between UPR and senescence [40]. Thus, it would be interesting to test if the prevention of senescent phenotype preserves proteostasis in ECs, such as those stimulated with AngII.

Prior studies have demonstrated that Drp1 likely mediates senescence through mitochondrial fission in cultured ECs [33] and cardiomyocytes [41]. Our data with mdivi1, together with these studies, suggest that mitochondrial fission is one potential mechanism by which AngII induces EC senescence. However, it should be noted that there is a statistically significant but slight decrease in EC numbers when comparing vehicle plus AngII to mdivi1 plus AngII. Since mdivi1 is known to act as a mitochondrial complex 1 inhibitor [42], it can be toxic to the cell when combined with AngII, which is a known inducer of mitochondrial ROS. Therefore, caution is prudent when considering the therapeutic utilization of mdivi1 as an anti-inflammatory and anti-aging reagent. Genetic approaches seem needed to clarify this issue in the future.

In human umbilical vein ECs, AngII causes senescence via activation of phosphatidyl-inositol 3 kinase [26]. A prior study also showed that Fos-related antigen 1 mediates AngII-induced senescence via induction of p16^INK4A^ in rat aortic ECs [43]. Thus, it is interesting to speculate on the upstream and downstream roles of phosphatidyl-inositol 3 kinase and Fos-related antigen 1 in Drp1 mediated mitochondrial fission and senescence in ECs under AngII activation, respectively. Regarding the signaling mechanism by which AngII elevates Drp1 activity and mitochondrial fission in ECs, it is likely that an upstream kinase phosphorylates and activates Drp1 in response to AT1R activation as demonstrated in VSMCs [16]. Alternatively, we have demonstrated that there is an inter-dependent relationship between Drp1 and the nuclear factor κ-B cascade in ECs, which mediates tumor necrosis factor α-induced EC inflammation [44]. In VSMCs, mdivi1 attenuated AngII-induced THP-1 adhesion as well as enhanced oxygen consumption [45]. Thus, it would be interesting to explore the relationship between Drp1, the nuclear factor κ-B cascade, and metabolic alterations in ECs upon AngII stimulation further. Lastly, the baseline and AngII-induced senescence seem exaggerated by passaging in some, but not all, experiments likely due to the combined effects of replicative senescence and stress (AngII)-induced premature senescence. Nonetheless, limited experimental *n* numbers is one of the limitations of this study.

In conclusion, pharmacological protection against ER stress, as well as inhibition of mitochondrial fission, prevented senescence and enhancement of monocyte adhesion in ECs stimulated by AngII. Removal of senescent ECs by a senolytic also mitigated monocyte adhesion. These findings suggest the potential roles for mitochondrial fission and ER stress in mediating endothelial inflamm-aging phenotype upon AngII exposure.

## 4. Materials and Methods

### 4.1. Cell Culture

Primary aortic ECs (passage 4–8) derived from 6–8 week Sprague-Dawley rats purchased from Cell Biologics (RA-6052) were cultured in Dulbecco’s modified Eagle’s medium (DMEM) with 1 g/L D-glucose, 1 mmol/L sodium pyruvate, 100 IU penicillin, 100 μg/mL streptomycin, and 10% fetal bovine serum (FBS) supplementation. Approximately 80–90% confluent cells serum-starved for 24 h on 12-well culture plates (growth area 3.8 cm^2^) were stimulated with AngII (100 nM) up to 48 h. For the 48 stimulations, the AngII stimulation was repeated at the 24 h time point. For pharmacological intervention, cells serum-starved for 24 h were incubated with fresh DMEM for 1 h, then pre-treated with indicated drugs or vehicle controls prior to stimulation with AngII. Pilot experiments in ECs with AngII stimulation (0, 1, 3, 6, 24, and 48 h time points) demonstrated that maximal mitochondrial fission induced by AngII occurred at 3 h, whereas consistent induction of THP-1 adhesion and senescence required 48 h stimulation with AngII, which set the time points in these assays. Key reagents and resources used in cell culture studies were described in Supplementary Table S1. 

### 4.2. Senescence-Associated β Galactosidase Assay

Senescence-associated β galactosidase activity was measured using the X-Gal staining system (Goldbio). In 12-well plates (growth area 3.8 cm^2^), at least 2 wells were assigned for each experimental condition. Upon AngII stimulation for 48 h, ECs were fixed with a 4% paraformaldehyde solution (Electron Microscopy Sciences) in phosphate-buffered saline (PBS) for 10 min, washed with Hanks Balanced Salt solution (HBSS) four times for 10 min, and incubated with 40 mM phosphate buffer containing 1 mg/mL X-Gal in 150 mM NaCl, 2 mM MgCl_2_, 5 mM K_3_Fe(CN)_6_, and 5 mM K_4_Fe(CN)_6_ at 37 °C for 12 h. Cells were washed twice with HBSS containing calcium and magnesium, and counter stained with Hoescht33342 (ThermoFisher, Waltham, MA, USA) for 5 min to stain nuclei. EC cultures were examined at 10× magnification to identify β galactosidase positive cells. Only blue-stained cell(s) clearly surrounded by the non-stained cell membrane structure were counted as positive cells. While the examiners were not blinded from the conditions, β galactosidase positive cells were counted in 5 randomly selected high-power fields (HPF) per condition and values were expressed as fold difference from 3–4 independent experiments (Figure 1, Figure 2 and Figure 4) or as percent difference in each experiment (Supplementary Figures) of positive cells versus total cell number. Total cell numbers were also demonstrated per condition in Figure 1, Figure 2, and Figure 4 as percent difference to the basal control conditions.

### 4.3. Leukocyte Adhesion Assay

To observe leukocyte attachment to ECs, THP-1 adhesion assay was performed, as described [5]. THP-1 monocytes (American Type Culture Collection) cultured in Roswell Park Memorial Institute/RPMI 1640 medium with 10% FBS, penicillin, and streptomycin were suspended in serum-free DMEM with 0.2% BSA and 5 μg/mL Hoechst 33342 for 30 min at 37 °C. These cells (10^4^ cells per cm^2^) were then applied to EC cultures on 24-well plates (growth area 1.9 cm^2^) for 30 min at 37 °C. EC cultures were then washed in PBS once for 5 min to remove unattached cells and subsequently imaged using a fluorescent inverted microscope. Stained THP-1 nuclei were counted to quantify adhesion to ECs as follows. Four wells were assigned for each experimental condition. Five randomly selected HPFs were captured per condition using a 10× objective lens. Images were imported into ImageJ, where the image was processed for background subtraction and conversion to a binary image followed by an analysis of particle count per visual field. Data were reported as fold difference to the control baseline mean value and compared across groups.

### 4.4. Mitochondrial Morphology Analysis

ECs were cultured on coverslips (growth area 2.54 cm^2^) fit on 12-well plates. Cells were infected with 10 moi adenovirus encoding mito-dsRed for 48 h prior to the treatment with the inhibitors and AngII. Upon AngII treatment for 3 h, ECs were washed twice for 5 min with DMEM and fixed in 3.7% paraformaldehyde for 15 min at 37 °C. Cells were then washed 3 times for 2 min with PBS and permeabilized with 0.2% Triton X-100 (Sigma T8787, Sigma, Saint Louis, MO, USA) in PBS for 10 min. Cells were washed 3 times for 2 min in PBS and mounted on glass slides with ProLong Gold Antifade (Thermo Fisher P36931). Fluorescent signals were detected on a 60× oil objective lens with a 1.5× adjustment attached to Olympus IX81 inverted fluorescent microscope with a Photometrics Cool SNAP HQ camera. Images were acquired using Metamorph software and imported into ImageJ, processed using iterative deconvolution, followed by rolling ball background subtraction and conversion to a color image for analysis using the Mito-Morphology plugin 5. The mitochondrial fission count (MFC) is defined as the number of mitochondria per cell (numerator) divided by the total mitochondrial area (pixels) of a given cell (denominator). Increasing MFC correlates to more mitochondria per given area, suggesting increased fission. Three wells were assigned for each experimental condition. Five randomly selected HPFs were captured per condition. In total, 3 independent experiments were used for analysis.

### 4.5. Statistical Analysis

Data are presented as mean ± SEM. Comparisons were performed via *t*-test for 2 groups, or via 1-way ANOVA with the post hoc Tukey method for multiple groups using Prism software (GraphPad, San Diego, CA, USA). Differences were considered statistically significant at *p* < 0.05.

## Supplementary Materials

The following are available online at https://www.mdpi.com/1422-0067/21/9/3112/s1, Figure S1: Effects of ABT737 and 4-PBA on AngII-induced EC senescence and effect of Olmesartan on AngII-induced THP-1 cell adhesion to ECs, Table S1: Key Reagents.

## Abbreviations

AngII	Angiotensin II
AT1R	Angiotensin II type 1 receptor
DMEM	Dulbecco’s Modified Eagle’s Medium
DMSO	Dimethyl sulfoxide
Drp1	Dynamin-related protein 1
ECs	Endothelial cells
ER	Endoplasmic reticulum
FBS	Fetal bovine serum
HPF	High power field
Mdivi1	Mitochondrial division inhibitor-1
MFC	Mitochondrial fragmentation count
PBS	Phosphate buffered saline
4-PBA	4-Phenylbutyrate
RAS	Renin angiotensin system
ROS	Reactive oxygen species
SASP	Senescence associated secretory phenotype
UPR	Unfolded protein response
VSMCs	Vascular smooth muscle cells
