# Supplementary material for: Involvement of Senescence and Mitochondrial Fission in Endothelial Cell Pro-Inflammatory Phenotype Induced by Angiotensin II
Source: Int J Mol Sci. 2020 Apr 28;21(9):3112. doi: 10.3390/ijms21093112 (PMC7247685; doi:10.3390/ijms21093112)
Supplement: Supplementary file 1 [file ijms-21-03112-s001.pdf]

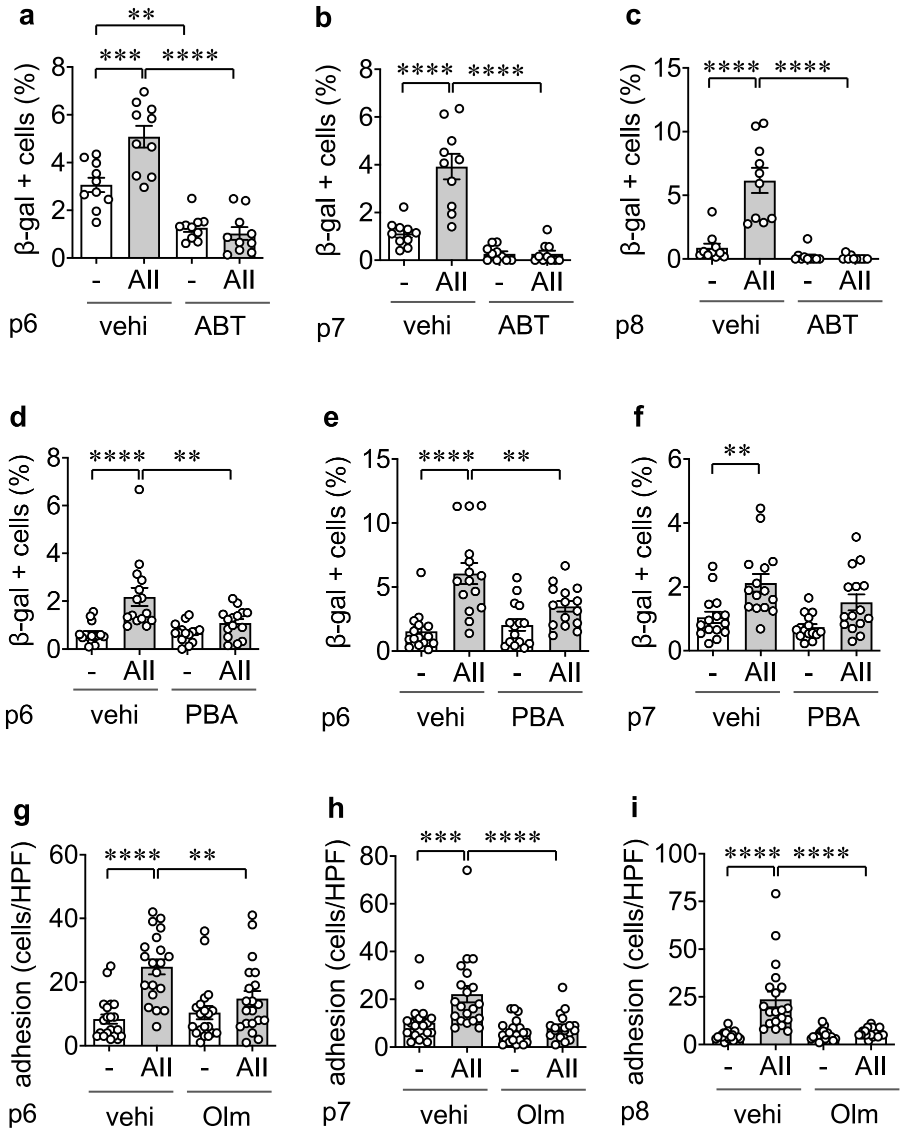

Figure S1. Effects of ABT737 and 4-PBA on AngII-induced EC senescence and effect of Olmesartan on AngII-induced THP-1 cell adhesion to ECs. (a, b and c) rat aortic ECs pretreated with a senolytic drug, ABT737 (ABT, 30 nM), or vehicle (vehi, 0.1% DMSO final) for 30 min were stimulated with 100 nM AngII (AII) for 48 hours. β galactosidase positive cells in 10 HPFs were counted in each group and expressed as % senescent cells per total cells. (d, e and f) rat aortic ECs pretreated with chemical ER chaperone, 4-PBA (PBA, 1 mM) or vehicle (vehi, PBS 0.1% final) were stimulated with 100 nM AngII (AII) for 48 hours. β galactosidase positive cells in 15 HPFs were counted in each group and expressed as % senescent cells per total cells. (g, h and i) Serum starved rat aortic ECs pretreated with AT1R antagonist, Olmesartan (Olm, 10 μM) for 30 min were incubated with 100 nM AngII (AII) for 48 hours. ECs were then incubated with THP-1 cells for 30 min, washed and adherent THP-1 cells were quantified. Attached THP-1 cells in 20 HPFs were counted in each group and expressed as cells per HPF. The bars in the graphs show the mean ± SEM. ** indicates $p<0.01$. *** indicates $p<0.001$. **** indicates $p<0.0001$.

Supplemental Table S1

Key Reagents

Name	*Source*	*Catalog #*
ABT737	Cayman	11501
Ad-mito-dsRed2	SignaGen Lab	SL100744
angiotensin II	Sigma	A9525
Hoechst 33342	Invitrogen	H3570
mdivi1	Med Chem Exp	HY-15886
Olmesartan/RNH6270	Sankyo	
X-Gal stain	GoldBio	X4281L
4-phenylbutyrate	Scandinavian Formulas	
